# Work-related tuberculosis in non-hospital sectors: social security evidence and emerging risks

**DOI:** 10.47626/1679-4435-2026-1571

**Published:** 2026-06-02

**Authors:** Isabella Valentini Ferreira, João Silvestre Silva-Junior

**Affiliations:** 1 Departamento de Medicina Legal, Bioética, Medicina do Trabalho e Medicina Física e Reabilitação, Faculdade de Medicina, Universidade de São Paulo, São Paulo, SP, Brazil

**Keywords:** tuberculosis, occupational exposure, work.

## Abstract

**Introduction:**

Tuberculosis is an infectious disease of major public health concern. Although most studies focus on health care professionals, other economic sectors may also present conditions that favor transmission and illness.

**Objectives:**

To analyze the occurrence of work-related social security benefits granted for tuberculosis in Brazil and to review scientific evidence on economic activities and occupations associated with increased risk of infection, excluding health care professionals.

**Methods:**

This ecological study used publicly available data from the Brazilian National Institute of Social Security. Work-related temporary disability benefits (B91) granted from 2022 to 2024 and linked to codes A15 to A19 of the International Classification of Diseases, 10th revision, were included. Results were described according to the number of benefits and the corresponding codes of the National Classification of Economic Activities. In parallel, a narrative literature review was conducted using PubMed and SciELO databases, including primary studies in Portuguese and English involving workers from different productive sectors.

**Results:**

A total of 115 B91 benefits due to tuberculosis were granted in 2022, 123 in 2023, and 65 in 2024. The literature identified increased tuberculosis risk among workers in cleaning, laundry, baking, construction, waste collection, sewage, textile manufacturing, and mining, associated with factors such as inadequate ventilation, silica exposure, unsanitary conditions, and social vulnerability.

**Conclusions:**

Work-related tuberculosis extends beyond the health care sector, affecting multiple productive sectors. Recognizing these exposures is essential to guide surveillance, inspection, and prevention measures aimed at reducing occupational tuberculosis.

## INTRODUCTION

Tuberculosis (TB) is an infectious disease transmitted via the airborne route and most commonly caused by *Mycobacterium tuberculosis* (also known as Koch’s bacillus). In rare cases, it may be caused by other related species, such as *M. bovis, M. africanum,* and *M. microti*. The disease primarily affects the lungs, although it may also involve other organs, including the kidneys, meninges, and bones [^1^].

The relationship between TB and occupation can be broadly classified into 3 categories [^2^]:

1. Occupations involving workers at high risk for TB, such as those in less favorable socioeconomic conditions: unskilled and low-paid workers [^2^].
2. Occupations that increase susceptibility to infectious organisms: workers predisposed due to silicosis (e.g., those employed in mining, quarrying, foundry, and ceramic manufacturing) [^2^].
3. Occupations that increase the chance of exposure to infection in environments conducive to transmission: workers in hospitals, mycobacteriology laboratories, and autopsy rooms [^2^].

In general, occupational TB infection or disease is easier to define than to document. Brazilian data on the occupational status of reported TB cases do not distinguish infections acquired in the workplace from those acquired in the community [^3^]. In this context, an “outbreak” may be defined as the transmission of

*M. tuberculosis* that results in infection or active disease among workers, patients, and other exposed individuals in health care facilities, correctional facilities, or other settings in which individuals with TB are treated, assisted, or detained [^3^].

Ordinance GM/MS No. 1,999 issued on November 27, 2023, by the Brazilian Ministry of Health, is the most up-to-date document presenting the List of Work-Related Diseases. TB appears on the list in its different clinical forms, corresponding to codes A15 to A19 of the International Classification of Diseases, 10th revision (ICD-10), supporting the presumed association between TB and work [^4^].

The Social Security Epidemiological Technical Nexus (Nexo Técnico Epidemiológico Previdenciário, NTEP) was established by Law No. 11,430, enacted on December 26, 2006 [^5^], which allows medical examiners of the Brazilian National Institute of Social Security (Instituto Nacional de Seguro Social, INSS) to recognize the occupational nature of disability based on the existence of an epidemiological technical nexus between work activities and the health condition causing disability, according to the relationship between the company’s economic activity and the corresponding disease listed in the ICD-10 [^5^]. Therefore, ICD-10 codes A15 to A19 are associated, under the NTEP framework, with specific codes of the National Classification of Economic Activities (Classificação Nacional de Atividades Econômicas, CNAE), as shown in Chart 1.

**Chart 1:** CNAE codes and their respective descriptions

0810 - Quarrying of stone and other raw materials for construction (building stone, marble, granite, sand, etc.)
1091 - Production of bakery products
1411 - Manufacture of underwear
1412 - Manufacture of wearing apparel, excluding underwear
1533 - Manufacture of footwear
1540 - Manufacture of parts of footwear
2330 - Manufacture of other concrete, cement, fiber cement, plaster, and similar products
3011 - Construction of vessels and fluctuating structures
3701 - Management of sewage systems
3702 - Sewage-related activities, excluding sewage system management
3811 - Collection of non-hazardous waste
3812 - Collection of hazardous waste
3821 - Treatment and disposal of non-hazardous waste
3822 - Treatment and disposal of hazardous waste
3839 - Composting plants
3900 - Decontamination and other waste management services
4120 - Building construction
4211 - Construction of highways and railways
4213 - Urban development works - streets, public squares, and sidewalks
4222 - Construction of water supply networks, sewage systems, and related works
4223 - Construction of pipeline transportation networks: oil pipelines, gas pipelines, and slurry pipelines
4291 - Port, maritime, and inland waterway construction works
4299 - Construction of sports and recreational facilities
4312 - Drilling and probing
4321- Electrical installation and maintenance
4391 - Foundations works
4399 - Specialized construction services not elsewhere specified
4687 - Wholesale trade of waste and scrap
4711 - Retail sale of general merchandise, predominantly food products
4713 - Non-specialized retail sale without predominance of food products
4721 - Retail sale of dairy products and deli items
4741 - Retail sale of painting supplies
4742 - Retail sale of electrical materials
4743 - Retail sale of glass products
4744 - Retail sale of construction materials in general
4789 - Retail sale of other new products not elsewhere specified
4921 - Municipal scheduled road passenger transport
4923 - Motorcycle taxi services; motorcycle courier services; passenger transport services; self-employed driver; taxi or similar services provided by ride-hailing drivers
4924 - School transport services
4929 - Other road passenger transport not elsewhere specified
5611 - Restaurants and other food and beverage service establishments
7810 - Recruitment and placement of personnel
7820 - Provision of temporary labor
7830 - Provision and management of outsourced human resources
8121 - Building and residential cleaning services
8122 - Immunization and urban pest control
8129 - Sterilization, sanitization, and environmental disinfection
8610 - Emergency care activities in emergency departments and hospital units for urgent care
9420 - Labor union activities
9601 - Laundry services

CNAE = National Classification of Economic Activities (Classificação Nacional de Atividades Econômicas).

A substantial portion of the literature addresses the risk of TB among health care professionals, including physicians, nurses, nursing technicians, and caregivers. However, there is limited discussion regarding the risk among workers in other occupations.

The World Health Organization (WHO) estimates that, in 2022, approximately 105,000 individuals developed TB in Brazil, of whom 87,344 were diagnosed and treated. This corresponds to a case detection rate of 83%, representing a 9.5% increase compared with 2021, when the country reported a detection rate of 75.8% [^6^]. In 2022, TB remained the second leading cause of death worldwide due to a single infectious agent, surpassed only by COVID-19 [^6^].

Given the impact of this disease on public health in Brazil, a better understanding of the occupational settings in which preventive measures and other interventions may be implemented is necessary in order to reduce national TB incidence and prevalence rates and, consequently, TB-related morbidity and mortality.

This study aimed to discuss other occupations in which TB may be recognized as a work-related disease, excluding health care professionals, in order to address epidemiological measures targeted at these worker groups.

## METHODS

This ecological study was based on publicly available data from the INSS Infologo AEPS platform [^7^]. Benefits granted according to the following criteria were considered: ICD group related to TB and the main benefit categories, including the categories “sickness benefits” and “work-related temporary disability benefits.” Sickness benefits are granted when the disease or condition is not related to occupational risk exposures present in the workplace or working conditions, thus characterizing a social security benefit. In contrast, work-related temporary disability benefits(B91) are characterized by medical examiners when an epidemiological technical nexus is recognized, whether due to occupational accidents, commuting accidents, work-related diseases, and occupational diseases [^8^,^9^].

Chart 1 presents the CNAE codes that, according to the NTEP, establish an association with TB-related illness, as provided by Decree No. 3,048/1999, as well as their respective descriptions. CNAE code 8610 (emergency care activities in emergency departments and hospital units for urgent care) was excluded from the analysis because it is related to health care professionals, who were not the focus of this study.

Primary studies related to the topic involving workers other than health care professionals and published in English or Portuguese were included in the discussion. Searches were conducted in the MEDLINE (via PubMed) and SciELO databases. No restrictions were applied regarding study design. For analysis purposes, some CNAE codes were grouped according to similarity.

## RESULTS

According to data from the Infologo AEPS platform, the number of work-related temporary disability benefits (B91) related to ICD-10 codes A15 to A19 was 115 in 2022. In 2023, this number increased to 123 benefits, followed by a reduction to 65 in 2024.

With respect to CNAE codes, Table 1 presents the distribution of B91 benefits according to the company’s CNAE code associated with work leave.

**Table 1:** Number of work-related temporary disability benefits (B91), according to CNAE code (economic activity) and ICD-10, Brazil, 2022-2024

CNAE	Description of economic activity	A15	A16	A17	A18	A19
4711	Retail sale of general merchandise, predominantly food products	22	5	0	0	0
8121	Building and residential cleaning services	17	2	0	0	0
3811	Collection of non-hazardous waste	14	1	0	1	0
4120	Building construction	8	4	0	0	0
4921	Municipal scheduled road passenger transport	7	1	0	0	0
9420	Labor union activities	7	2	0	1	0
5611	Restaurants and other food and beverage service establishments	5	1	0	1	1
4929	Other road passenger transport not elsewhere specified	5	1	0	0	0
4721	Retail sale of dairy products and deli items	1	3	0	0	0
4744	Retail sale of construction materials in general	3	0	0	1	0
2330	Manufacture of other concrete, cement, fiber cement, plaster, and similar products	2	1	0	0	0
7820	Provision of temporary labor	1	2	0	0	0
4299	Construction of sports and recreational facilities	3	0	0	1	0
3702	Sewage-related activities, excluding sewage system management	3	2	0	0	0
1412	Manufacture of wearing apparel, excluding underwear	2	0	0	1	0
4399	Specialized construction services not elsewhere specified	2	0	0	0	0
4211	Construction of highways and railways	2	0	0	0	0
4687	Wholesale trade of waste and scrap	1	1	0	0	0
4789	Retail sale of other new products not elsewhere specified	1	1	0	0	0
1091	Production of bakery products	1	1	0	0	0
4391	Foundations works	0	1	0	0	0
4713	Non-specialized retail sale without predominance of food products	1	0	0	0	0
1411	Manufacture of underwear	1	0	0	0	0
4213	Urban development works - streets, public squares, and sidewalks	1	0	0	0	0
3011	Construction of vessels and fluctuating structures	2	0	0	0	0

B91 = work-related temporary disability benefits; CNAE = National Classification of Economic Activities (Classificação Nacional de Atividades Econômicas, ICD-10 = Internacional Classification of Diseases, 10th revision.

## DISCUSSION

TB is not only an individual health concern but also a potential source of outbreaks, particularly in collective environments such as restaurants, commercial areas, and nightclubs. Studies have shown that, in these environments, the presence of an infected individual (whether a worker or customer) may facilitate disease transmission through coughing. One study reported a significant outbreak in a nightclub, in which multiple cases were traced back to an initial source consisting of infected employees, highlighting the rapid spread of TB among customers and staff [^10^].

Studies have also suggested a higher incidence of pulmonary TB among bakers compared with other occupations. In one study involving 26 confirmed TB cases, 8 patients reported an occupational history of baking activities, indicating a possible association between this occupation and disease development [^11^]. Working conditions in bakeries may contribute to respiratory disorders and increase susceptibility to infections, such as TB, due to factors including:

- Inadequate ventilation: it may favor the accumulation of airborne particles, such as flour dust and other allergens, which can irritate the respiratory tract [^12^];
- Occupational exposure: inhalation of silica and carbon particles generated during cooking processes has been associated with bronchial anthracosis, a condition related to TB [^11^,^12^], potentially impairing lung function and defense mechanisms against infections;
- Poor hygiene conditions: unsanitary work environments may facilitate foodborne diseases, further compromising the health status of workers already vulnerable to respiratory diseases.

Ensuring adequate ventilation, proper hygiene practices, and nutritional support is essential for protecting the health of bakery workers.

Cleaning workers, especially those employed in health care settings, are exposed to a significant risk of TB infection. This group presents higher annual rates of tuberculin skin test conversion, indicative of latent pathogen infection. One study identified that housekeeping workers had an annual conversion rate of 6.9%, significantly higher than that observed in other health care professionals. Factors contributing to this increased risk included older age and work in environments with high patient turnover, which increases exposure to potentially infectious individuals [^13^].

The causative agent of TB is transmitted primarily through airborne particles expelled during coughing or sneezing by infected individuals. Among cleaning workers, the risk of infection increases with prolonged exposure to environments previously occupied by individuals with active TB. However, this risk may be substantially reduced when sufficient time elapses between the departure of the infected individual and the entry of the worker into the environment, particularly when adequate ventilation is ensured.

TB also represents a major public health concern among waste collectors and workers involved in the management of health care waste. These workers face increased health risks due to exposure to a variety of pathogens present in waste materials. One study demonstrated low adherence to the use of personal protective equipment (PPE), such as gloves, among these workers, which increases the risk of contracting diseases, including TB [^14^]. The nature of their occupational activities favors exposure to biological and mechanical vectors potentially capable of transmitting infections.

In a notable incident in Washington State, United States, 3 workers developed pulmonary TB after processing contaminated health care waste. Investigations revealed that the waste contained viable cultures of *M. tuberculosis* that had not been properly decontaminated prior to disposal. This incident underscores the potential for TB transmission in settings where hospital waste is inadequately managed [^15^].

In addition to TB, waste collectors and workers involved in the management of health care waste are at increased risk of developing respiratory diseases and other chronic conditions associated with exposures present in the workplace [^16^].

Although the primary route of TB transmission is airborne, there are specific considerations related to laundry services, particularly in health care settings where patients with TB receive care. Studies indicate that laundry workers, along with maintenance and cleaning personnel in health care facilities, are at a significantly higher risk of TB pathogen infection compared with nursing staff. This increased risk has been attributed to exposure to contaminated bed linens and inadequate ventilation in areas designated for the care of patients with TB [^17^].

Laundry workers may come into contact with bed linens used by patients with TB, which may represent a transmission risk if appropriate infection control measures are not implemented. In this context, it is essential that laundry services in health care settings adopt strict protocols for handling potentially contaminated materials. Effective infection control measures include the use of PPE, such as gloves and masks, during the handling of soiled linens, as well as washing materials with detergent at high temperatures to eliminate potentially present bacteria, including *M. tuberculosis*.

The CNAE codes 2330 (manufacture of other concrete, cement, fiber cement, plaster, and similar products), 4120 (building construction), 4211 (construction of highways and railways), 4213 (urban development works - streets, public squares, and sidewalks), 4299 (construction of sports and recreational facilities), 4312 (drilling and probing), 4391 (foundation works), and 4399 (specialized construction services not elsewhere specified) may be analyzed together due to similarities in the activities performed.

These economic activities share characteristics similar to those observed in mining work, particularly with respect to confined environments and inadequate ventilation. Furthermore, studies have demonstrated biofilm formation by pathogenic strains of *M. tuberculosis* on cement, ceramic, and stainless steel surfaces [^18^]. In general, biofilm formation is more pronounced on cement surfaces than on ceramic or stainless steel. This finding is particularly relevant in developing countries, where cement remains an important contact surface in daily occupational activities, including environments exposed to high temperatures, such as those in the construction industry.

Construction workers are at significantly increased risk of pulmonary TB, particularly those exposed to respirable silica dust. This risk is exacerbated in settings such as road construction sites, where temporary workers frequently perform activities without appropriate protective measures [^18^].

Electricians may also be exposed to the risk of TB in various occupational settings, especially when working in older buildings or facilities with inadequate ventilation. These environments may harbor individuals with undiagnosed active TB, thereby increasing the risk of transmission. Although there few studies specifically involving electricians, evidence indicates that maintenance and engineering personnel in health care facilities exhibit higher infection rates than those observed among clinical staff, suggesting that similar risks may apply to electricians working in health care settings or densely populated environments [^17^].

Public bus transportation may be considered a congregate setting, according to the WHO definition. This type of setting may significantly facilitate the transmission of TB, including multidrug-resistant and extensively drug-resistant forms, particularly under conditions of overcrowding and inadequate ventilation, such as when all vehicle windows remain closed during travel. Opening windows and ensuring adequate air circulation in confined or congregate spaces are recommended measures for the prevention of TB and other airborne infectious diseases [^19^].

In a study conducted in Peru, regular users of buses/minibuses were almost 12 times more likely to develop TB than non-users. Commuting time to work was not associated with TB in the multivariate analysis, suggesting that the type of transportation used may contribute to the dose-response relationship observed in the univariate analysis. These findings are consistent with previous studies conducted in Lima, Peru, which also identified an association between bus/minibus use and TB risk, even after adjustment for other known risk factors [^20^].

Health authorities emphasize the need for regular health screenings in restaurants and other commercial establishments as a measure to prevent outbreaks. The potential for TB transmission in these settings underscores the importance of monitoring and controlling infections among workers, particularly those with compromised immune systems or other risk factors [^10^].

Wastewater and sewage management involves activities performed in closed channels, underground galleries, and sewage treatment plants. These workers carry out tasks such as accessing manholes and operating sewage treatment facilities, exposing them to specific occupational hazards resulting from contact with chemical gases, bioaerosols, and microorganisms. The main routes of exposure and transmission of harmful agents derived from sewage include inhalation, oral ingestion (through splashes, contaminated food, and hand-to-mouth contact), and penetration through the skin or mucous membranes, particularly in the presence of skin lesions or improper use of PPE [^16^].

In addition to environmental risks, sewage workers are one of the most vulnerable and neglected socio-occupational groups in society. These professionals face social exclusion, economic constraints, and neglect by health care services due to the complex interplay of cultural and systemic factors. All of these factors also influence the health status, health care-seeking behavior, and therapeutic pathways of this occupational group [^16^]. TB is also a common disease among sewage workers.

In a study conducted in India involving 104 workers in this sector, 22 individuals (21.15%) were diagnosed with TB after testing. A higher frequency of the disease was observed among workers with longer occupational exposure. It is well established that changes in the pulmonary mucosa resulting from respiratory diseases, such as chronic obstructive pulmonary disease and smoking (conditions present in a significant portion of workers in the study), impair pulmonary defense mechanisms and increase susceptibility to airborne infections, including TB. Furthermore, the study highlighted the limited availability of PPE in the workplace and the low level of awareness regarding its importance [^21^].

Moreover, TB in sewage workers may be smear-negative for acid-fast bacilli (AFB), since fibrotic lung disease secondary to repeated inhalational exposure to harmful agents may develop early during occupational activity. This may result in delayed or incorrect diagnosis unless there is a high index of clinical suspicion [^21^].

An individual with TB not only suffers from the disease but also serves as a source of infection for others, including family members, friends, coworkers, and the wider community. Therefore, early and timely diagnosis not only benefits the patient but also contributes to primary prevention among at-risk populations in the community, including the prevention of multidrug-resistant cases [^21^].

Workers in the garment industry were evaluated in a study conducted in Qatar [^22^]. The results demonstrated that a very high proportion of workers in the assessed factories had TB (43%). This is a relevant finding because it not only represents a serious health consequence for individuals with TB but also indicates a risk of institutional transmission of TB in the factory environment. The high infection rate may be related to inadequate ventilation in factories and the high density of workers in confined spaces, factors that facilitate disease transmission from infected individuals. Furthermore, many factories and labor camps are located in industrial zones, areas with a higher prevalence of TB.

The extraction of stone and other raw construction materials is among the CNAE codes most frequently cited in the TB literature. Working conditions in mines are considered high risk for TB transmission due to exposure to silica and mineral dust, which may lead to the development of silicosis and consequently increase the risk of TB. Additionally, the general living conditions of miners, along with the migration processes characteristic of this activity, also favor the spread of TB in the community, with serious implications for disease prevention and control strategies [^23^].

In sub-Saharan Africa, for example, miners present some of the highest TB rates recorded among working populations worldwide, ranging from 3,000 to 7,000 cases per 100,000 miners annually in certain regions. The incidence of TB among miners is estimated to be up to 10 times higher than that observed in the populations from which these workers originate [^23^].

Occupational TB may occur in economic activities involving workers at high risk of infection, in occupations that increase susceptibility to infectious agents, and in occupations that increase the likelihood of exposure to infection in environments conducive to transmission [^2^].

Exposure to silica and mineral dust increases the risk of silicosis and bronchial anthracosis, conditions that predispose individuals to infection with *M. tuberculosis*. In addition, inadequate ventilation, prolonged stays in enclosed environments, and high worker density in confined spaces increase the risk of bacillus transmission among workers, especially in factories, mines, and water and sewage systems.

Economic activities that require worker migration also contribute to the transmission process, as these individuals may carry the bacillus between the workplace and their homes. Transmission is also more strongly associated with socially vulnerable groups, more industrialized areas, and occupations related to waste management and the cleaning of outpatient and hospital environments.

These findings may support the implementation of measures such as educating these working populations about the transmission route of *M. tuberculosis*, the importance of proper PPE use, and the correct disposal of contaminated materials. Furthermore, they reinforce the need for inspection of working conditions and the implementation of improvements in occupational environments, with the aim of reducing TB-related morbidity and mortality in the country.

Historically, TB has represented a significant occupational risk aboard ships. A study conducted on a vessel with 1,175 crew members documented 25 cases of active pulmonary TB over a 1-year period. The confined environment facilitated the rapid spread of the disease, highlighting the need for regular screening protocols and the isolation of symptomatic individuals [^24^].

Individuals involved in ship construction and repair are also classified as being at increased risk of developing TB. This risk is attributed to factors such as close physical proximity and potential exposure to infected individuals. One study reported that workers in this sector had higher odds ratios for TB than those in other occupations [^25^].

The NTEP also presents limitations, particularly due to its low specificity regarding workers’ occupations, as the classification is based on the company’s economic activity rather than on specific job functions. This represents a limitation of the present study, since occupational environments and risks may vary considerably among workers within the same company.

Some CNAE codes were not included in the results because no studies related to these economic activities were identified, such as codes 9420 (labor union activities) and 7810 (recruitment and placement of personnel). This suggests the need for further research in these areas regarding the topic.

## CONCLUSIONS

A substantial portion of the literature highlights the risk of TB acquisition among health care professionals, often to the exclusion of other occupational sectors. In the present study, we concluded that several economic sectors also warrant attention regarding TB. These findings may support the implementation of workplace inspection and prevention measures, such as improving ventilation, monitoring hygiene conditions, and promoting the appropriate use of PPE, in order to reduce the number of work-related TB cases.

## Data Availability

Upon publication, the data will be made available by the authors upon request.

## References

[1] Rolla V. (2013). Glossário de Doenças da Fundação Oswaldo
Cruz.

[2] Tam CM, Leung CC. (2006). Occupational tuberculosis: a review of the literature and the
local situation. Hong Kong Med J.

[3] Field MJ. (2001). Tuberculosis in the workplace. National Academy Press;.

[4] Brasil, Ministério da Saúde (2023). Portaria GM/MS No 1.999, de 27 de Novembro de 2023. Altera a Portaria de
Consolidação GM/MS nº 5, de 28 de setembro de 2017 para
atualizar a Lista de Doenças Relacionadas ao Trabalho (LDRT).

[5] Brasil, Presidência da República (2006). Lei Nº 11.430, de 26 de Dezembro de 2006. Altera as Leis nºs 8.213, de
24 de julho de 1991, e 9.796, de 5 de maio de 1999, aumenta o valor dos
benefícios da previdência social; e revoga a Medida
Provisória nº 316, de 11 de agosto de 2006; dispositivos das Leis nºs
8.213, de 24 de julho de 1991, 8.444, de 20 de julho de 1992, e da Medida
Provisória nº 2.187-13, de 24 de agosto de 2001; e a Lei nº 10.699,
de 9 de julho de 2003.

[6] Brasil, Ministério da Saúde (2023). Brasil recupera índice de detecção
detuberculoseeaperfeiçoatratamento.

[7] Brasil, Infologo AEPS (2024). Base de Dados Históricos da Previdência Social.

[8] Brasil (1991). Lei No 8.213, de 24 de Julho de 1991. Dispõe sobre os Planos de
Benefícios da Previdência Social e dá outras
providências.

[9] Brasil, Presidência da República (1999). Decreto No 3.048, de 6 de Maio de 1999. Aprova o Regulamento da
Previdência Social, e dá outras providências.

[10] Gronauer W. (1994). [Employees and regular customers of restaurants and discos as
transmitters of tuberculosis]. Gesundheitswesen.

[11] Ghanei M, Aslani J, Peyman M, Asl MA, Pirnazar O. (2011). Bronchial Anthracosis: A Potent Clue for Diagnosis of Pulmonary
Tuberculosis. Oman Med J.

[12] Bakeries (2011). Food Processing Sectors.

[13] Sherman HA, Karakis I, Heimer D, Arzt M, Goldstein W, Bouhnik L (2011). Housekeeping health care workers have the highest risk for
tuberculin skin test conversion. Int J Tuberc Lung Dis.

[14] Dall’Agnol CM, Fernandes FS. (2007). Health and self-care among garbage collectors: work experiences
in a recyclable garbage cooperative. Rev Lat Am Enfermagem.

[15] Johnson KR, Braden CR, Cairns KL, Field KW, Colombel AC, Yang Z (2000). Transmission of Mycobacterium tuberculosis from medical
waste. JAMA.

[16] Arora VK, Chandra K, Chandra M. (2019). Occupational tuberculosis in sewage workers: A neglected
domain. Indian J Tuberc.

[17] Field MJ. (2001). Tuberculosis in the workplace.

[18] Adetunji V, Kehinde A, Bolatito O, Chen J. (2014). Biofilms formed by Mycobacterium tuberculosis on cement, ceramic,
and stainless steel surfaces and their controls. J Food Prot.

[19] Gebrehiwot TT, Tesfamichael FA. (2017). Knowledge, risk perception and practice regarding tuberculosis
transmission among long distance bus drivers in Addis Ababa, Ethiopia: a
cross sectional study. Ethiop J Health Sci.

[20] Zamudio C, Krapp F, Choi HW, Shah L, Ciampi A, Gotuzzo E (2015). Public transportation and tuberculosis transmission in a high
incidence setting. PLoS One.

[21] Chandra K, Arora VK. (2019). Tuberculosis and other chronic morbidity profile of sewage
workers of Delhi. Indian J Tuberc.

[22] Al-Khal AL, Bener A, Enarson DA. (2005). Tuberculosis among garment workers in an Arabian developing
country: State of Qatar. Arch Environ Occup Health.

[23] Stuckler D, Basu S, McKee M, Lurie M. (2011). Mining and risk of tuberculosis in sub-Saharan
Africa. Am J Public Health.

[24] Hardy MA. (1968). Epidemiology of Tuberculosis Aboard a Ship. JAMA.

[25] Nardell EA. (2016). Transmission and Institutional Infection Control of
Tuberculosis. Cold Spring Harb Perspect Med.

